# Upregulation of USP22 and ABCC1 during Sorafenib Treatment of Hepatocellular Carcinoma Contribute to Development of Resistance

**DOI:** 10.3390/cells11040634

**Published:** 2022-02-11

**Authors:** Yung-Sheng Chang, Chien-Wei Su, San-Chi Chen, Yen-Ying Chen, Yuh-Jin Liang, Jaw-Ching Wu

**Affiliations:** 1 Institute of Clinical Medicine, National Yang Ming Chiao Tung University, Taipei 11221, Taiwan; yschang2021@nycu.edu.tw (Y.-S.C.); scchen16@vghtpe.gov.tw (S.-C.C.); 2 Faculty of Medicine, School of Medicine, National Yang Ming Chiao Tung University, Taipei 11221, Taiwan; cwsu2@vghtpe.gov.tw; 3 Division of Gastroenterology and Hepatology, Department of Medicine, Taipei Veterans General Hospital, Taipei 11217, Taiwan; 4 Department of Oncology, Taipei Veterans General Hospital, Taipei 11217, Taiwan; 5 Department of Pathology, Taipei Veterans General Hospital, Taipei 11217, Taiwan; yychen26@vghtpe.gov.tw; 6 Medical Research Department, Taipei Veterans General Hospital, Taipei 11217, Taiwan; yjliang@vghtpe.gov.tw; 7 Cancer Progression Research Center, National Yang Ming Chiao Tung University, Taipei 11221, Taiwan

**Keywords:** sorafenib resistance, USP22, ABCC1, hepatocellular carcinoma

## Abstract

Sorafenib is a small molecule that blocks tumor proliferation by targeting the activity of multi-kinases for the treatment of advanced hepatocellular carcinoma (HCC). Increasing sorafenib resistance following long-term treatment is frequently encountered. Mechanisms underlying sorafenib resistance remain not completely clear. To further understand the mechanism of sorafenib resistance in HCC, we established sorafenib-resistant cell lines by slowly increasing sorafenib concentration in cell culture medium. Upregulation of USP22 and ABCC1 were found in Sorafenib-resistant cells. Sorafenib-resistant cells treated with USP22 siRNA showed significant reduction in endogenous mRNA and protein levels of ABCC1. During sorafenib treatment, upregulation of USP22 increases ABCC1 expression and subsequently contributes to sorafenib resistance in HCC cells. Immunohistochemical analysis revealed a positive correlation between USP22 and ABCC1 expression in tissue samples from sorafenib-resistant patients (Pearson’s correlation = 0.59, *p* = 0.03). Our findings indicate that upregulation of USP22 and ABCC1 expression during treatment contribute to sorafenib resistance in HCC cells and that USP22 has strong potential as a therapeutic target for overcoming sorafenib resistance in HCC patients.

## 1. Introduction

Hepatocellular carcinoma (HCC) is a highly malignant tumor with very poor prognosis. It is the fourth leading cause of cancer mortality worldwide and accounts for >700,000 deaths every year. Selection of treatment for HCC is based on tumor stage, liver functional reserve, and performance status [1]. Recommended treatment modalities are curative therapies (e.g., surgical resection, local ablation therapy, and liver transplantation) at early stage, transarterial chemoembolization (TACE) at intermediate stage, and systemic therapy at advanced stage (characterized by major vascular invasion or extrahepatic metastasis). In recent years, stereotactic body radiotherapy (SBRT) has also been recognized as a feasible and well-tolerated standard treatment option for patients with intermediate or advanced HCC [2]. Overall, curative therapies provide median survival time >5 years for patients with early-stage HCC; however, the benefit of surgery or ablation therapy is offset by a high rate of postoperative recurrence (up to 70%) [3]. Many HCC patients experience tumor progression to advanced stage following curative therapy or TACE. Such patients are often recommended to receive systemic therapy, e.g., molecular target therapy or immune checkpoint inhibitors. Sorafenib was the first drug that obtained FDA approval for first-line systemic therapy of unresectable HCC in 2007. Sorafenib is a multi-targeted small molecule that inhibits activities of VEGF, PDGF, EGF, and Raf, thereby blocking tumor proliferation and angiogenesis. Unfortunately, HCC patients often discontinue sorafenib treatment because of the appearance of serious adverse effects, or resumption of tumor progression resulting from development of drug resistance [4]. To improve outcomes of such HCC patients, we need to elucidate the mechanism of sorafenib resistance with a purpose of developing novel compounds that overcome the resistance.

Cancer is a complex disease and typically involves dysregulation of various cancer-related genes and pathways. Many small molecules or compounds have been discovered and synthesized for specific targeting in cancer therapy. This type of cancer therapy has several limitations [5,6]. Upregulation of multidrug-resistant proteins is associated with development of drug resistance in cancer cells [7,8]. Suppression of multidrug-resistance genes increases drug sensitization in cancer cells [9,10]. Long-term exposure of HCC cells to sorafenib induces drug resistance and epithelial-mesenchymal transition [11]. Sorafenib resistance in HCC cells has been correlated with increased stemness [12].

Protein ubiquitination (addition of ubiquitin molecules to Lys residues) is involved in regulation of many essential cellular processes. Protein ubiquitination levels are controlled by ubiquitylating and deubiquitylating enzymes. Ubiquitin-specific proteases (USPs) are the largest family of deubiquitinases, and ~56 USPs are present in humans. Aberrant expressions of USPs often show a strong correlation with cancer progression and USPs have potential application as novel targets for cancer therapy [13,14,15,16]. For example, USP22 was shown to mediate multidrug resistance of fluorouracil (5-FU) through activation of Sirtuin 1 (SIRT1) and Akt signaling pathway, and high USP22 expression was associated with poor prognosis in HCC [17]. The role of USP22 in sorafenib resistance has not been investigated.

To further understand the mechanism of sorafenib resistance in HCC, we established sorafenib-resistant cell lines by slowly increasing sorafenib concentration in cell culture medium. Compared with parental cell line, we found that USP22 and ABCC1 was highly expressed in sorafenib resistant cells. USB22 has been reported to promote ABCC1 expression in HCC cells by activating the SIRT1/Akt/MRP1 pathway. However, the relationship between USP22, ABCC1 and sorafenib resistance has not been reported. The purpose of this study was to further clarify the relationship between USP22 and ABCC1 and its role on sorafenib resistance of HCC.

## 2. Materials and Methods

### 2.1. HCC Patients

Immunohistochemical (IHC) staining of ABCC1 and USP22 in HCC specimens obtained from Biobanks of Taipei Veterans General Hospital was analyzed retrospectively under IRB approval. IHC staining results were correlated with sorafenib resistance and treatment response.

### 2.2. Cell Culture and Drug Treatment

Cell lines Tong/HCC, Hep3B, Huh7, Mahlavu, SNU-387 and SNU-449 were maintained in 100-mm tissue culture dishes in DMEM (Life Technologies Corp.; Carlsbad, CA, USA) supplemented with 10% FBS (Biological Industries USA; Cromwell, CT, USA) and 100 U/mL each of penicillin-streptomycin (Gibco BRL; Grand Island, NY, USA), L-glutamine (Gibco) and nonessential amino acids (Gibco) at 37 °C in 5% CO_2_ atmosphere. Cells were seeded and incubated in 60-mm tissue culture dishes or 96-well, flat-bottom plates in DMEM/10% FBS for 24 h, then treated with various dosages with sorafenib at 37 °C in 5% CO_2_ atmosphere for 48 or 72 h.

### 2.3. Cell Viability Analysis

Cells (1000-5000 per well) were seeded in triplicate and incubated in 96-well, flat-bottom plates in DMEM/10% FBS for 18 h, and treated with sorafenib for 72 h and read on ELISA plate reader. Medium was then replaced with medium containing MTS reagent (Promega Corp.; San Luis Obispo, CA, USA), and plates were incubated at 37 °C in 5% CO_2_ atmosphere for optimal period and read on ELISA plate reader at wavelength 490 nm.

### 2.4. Establishment of Sorafenib-Resistant HCC Cells

HCC cells were subjected to steadily increasing medium concentrations of sorafenib starting at 3.5–10.8 μM sorafenib (IC50 for HCC cells), and dose increasing by 0.5 μM each week. It usually takes 3 months to establish sorafenib-resistant HCC cells. The highest concentration of sorafenib is 10.83 μM for SNU-449 cells and 7.17 μM for Hep3B cells during the selection period. The final concentration of sorafenib is 8.33 μM for SNU-449 cells and 3.67 μM for Hep3B cells in culture medium while sorafenib-resistant HCC cells are maintained.

### 2.5. Real-Time Quantitative Reverse-Transcription Polymerase Chain Reaction (RT-qPCR)

Total RNA from cells was extracted using TRIzol reagent (Thermo Fisher Scientific; Carlsbad, CA, USA) and reverse-transcribed using SuperScriptTM IV first-strand synthesis system (Thermo Fisher). RT-qPCR was performed using iTaq Universal SYBR Green Supermix (Bio-Rad Laboratories; Hercules, CA, USA) on QuantStudioTM 3 Real-Time PCR System (Thermo Fisher). The following primers were used: for USP22: forward 5′-ATATTCACGAGCATGCGAAG-3′, reverse 5′-GGTTGGTTCCCAAGTTGAAA-3′; for GAPDH: forward 5′-AGAAGGCTGGGGCTCATTTG-3′, reverse 5′-AGGGGCCATCCACAGTCTTC-3′; for ABCC1: forward 5′-ACCCTAATCCCTGCCCAGAG-3′, reverse 5′-CGCATTCCTTCTTCCAGTTC-3′; for SIRT1: forward 5′-TGCTGGCCTAATAGAGTGGCA-3′, reverse 5′-CTCAGCGCCATGGAAAATGT-3′. Data were analyzed by 2^−ΔΔCq^ method.

### 2.6. Protein Extraction and Western Blot Analysis

Cells were scraped off dishes and lysed in NET buffer containing 50 mM Tris-HCl (pH 7.4), 150 mM NaCl, 1 mM EDTA, 1% Triton X-100, 1% deoxycholate, and 0.1% SDS. Isolated proteins were separated by SDS-PAGE and blotted onto PVDF membranes. Expression of the molecules listed below was analyzed by incubation of membrane with the indicated primary antibody at 4 °C overnight, followed by secondary antibody. ABC transporters: anti-ABCC1 (1:1000; #72202, Cell Signaling Technology; Danvers, MA, USA [CST]). USP22: anti-USP22 (1:1000; ab195289, Abcam; Cambridge, MA, USA). USPs: USP antibody sampler kit (1:1000; #12894, CST). SIRT1: anti-SIRT1 (1:1000; #9475, CST). Akt: anti-phospho-Akt and anti-Akt (1:1000; #2965/#4060/#4691, CST). Erk: anti-phospho-Erk and anti-Erk (1:1000; #4370/#4695, CST). As reference protein: heat shock cognate protein (Hsc70): mAb HSC70 (B-6) (1:5000; Santa Cruz Biotechnology; Dallas, TX, USA). Signals were developed using western blot chemiluminescence reagent (Millipore Corp.; Burlington, MA, USA) and detected with X-ray film.

### 2.7. Establishment of shRNA-Bearing Cells

Bacterial clones and customized pseudo-typed lentivirus bearing shRNA were from RNAi core facility, Academia Sinica, R.O.C. 600,000 target cells were seeded onto 6-cm petri dishes, and viruses were added the next day in the presence of 8 μg/mL polybrene. 1–3 days later, virus-containing medium was removed, and medium containing 2.5 μg/mL puromycin was added for selection of infected cells. After 48 h, a knockdown cell line was established, and knockdown efficiency was confirmed by RT-qPCR and western blotting.

### 2.8. Statistical Analysis

Data were analyzed by one-way analysis of variance (ANOVA) followed by post hoc tests for multiple comparisons. Student’s *t*-test was applied for two-group comparisons. Values are presented as mean ± SD. Differences with *p* < 0.05 are considered statistically significant.

## 3. Results

### 3.1. Responses to Sorafenib Treatment Correlated with the Grading and Invasiveness of HCC Cell Lines

**Figure 1:**
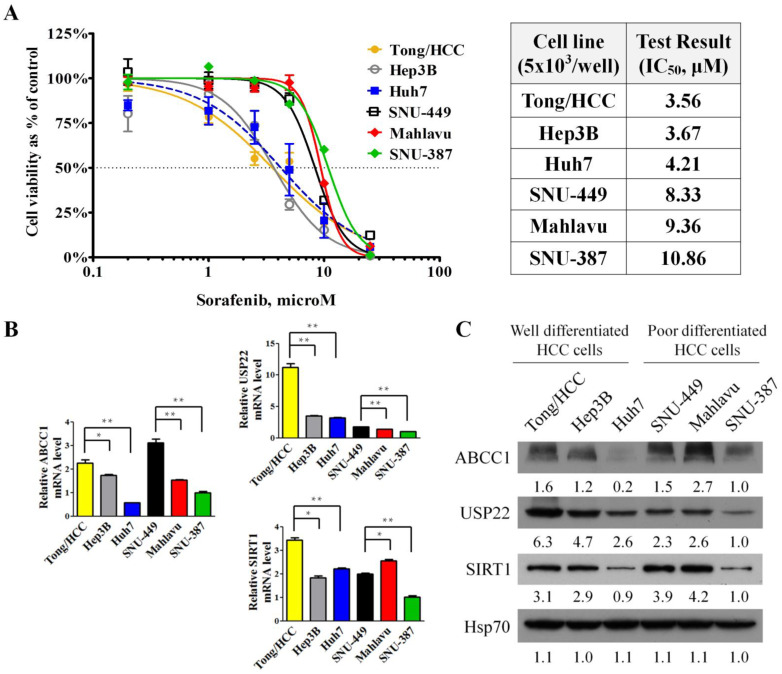
Growth-inhibitory effects of sorafenib in HCC cells: (**A**) Cells were treated with various concentrations of sorafenib (0.2, 1, 2.5, 5, 10, 25 μM) for 72 h. Antiproliferative effects of sorafenib on HCC cell lines were determined by MTT assay (**left panel**) and IC_50_ value of HCC cell lines to sorafenib (**right panel**). (**B**,**C**) Expression of ABCC1, USP22, and SIRT1 in HCC cell lines analyzed by RT-qPCR and western blotting. Numeric values listed below the bands represent the indicated protein expression by densitometry analysis relative to levels in SNU-387 cells. *: *p* < 0.05; **: *p* < 0.01 compared with indicted HCC cells.

The effects of sorafenib on the growth of different HCC cell lines were evaluated (Figure 1A). Mahlavu, SNU-387, and SNU-449 were classified as poorly differentiated cell lines [18]. Tong/HCC, Hep3B, and Huh7 were classified as well differentiated cell lines [19]. HCC cells were cultured and treated with sorafenib at concentrations ranging from 0.2 to 25 μM for 72 h. Mahlavu, SNU-387, and SNU-449 with high-grade malignancy were relatively more resistant to sorafenib, whereas Tong/HCC, Hep3B, and Huh7 were relatively more sensitive. Thus, HCC grading and invasiveness correlated with sorafenib resistance.

USP22 was shown to play a critical role in the development of 5-FU chemoresistance in HCC cells and increases the expression of ABCC1 via interacting with SIRT1. To further evaluate relationships among USP22 and sorafenib resistance, expression of ABCC1, USP22, and SIRT1 in 6 HCC cell lines were analyzed by RT-qPCR and western blotting (Figure 1B,C). Positive correlation among USP22, SIRT1, and ABCC1 expression were observed in all 6 HCC cell lines. However, the expression of ABCC1, USP22, and SIRT1 were consistently maintained at relatively low basal level in cells more resistant to sorafenib (Huh7 or SNU-387) at different group (well differentiated and poorly differentiated cell lines). Results indicated that the sensitivity to sorafenib in normal culture conditions may be due to cell inherent characteristics, but not the basal levels of USP22 and ABCC1.

### 3.2. Establishment of Sorafenib-Resistant Cell Lines

**Figure 2:**
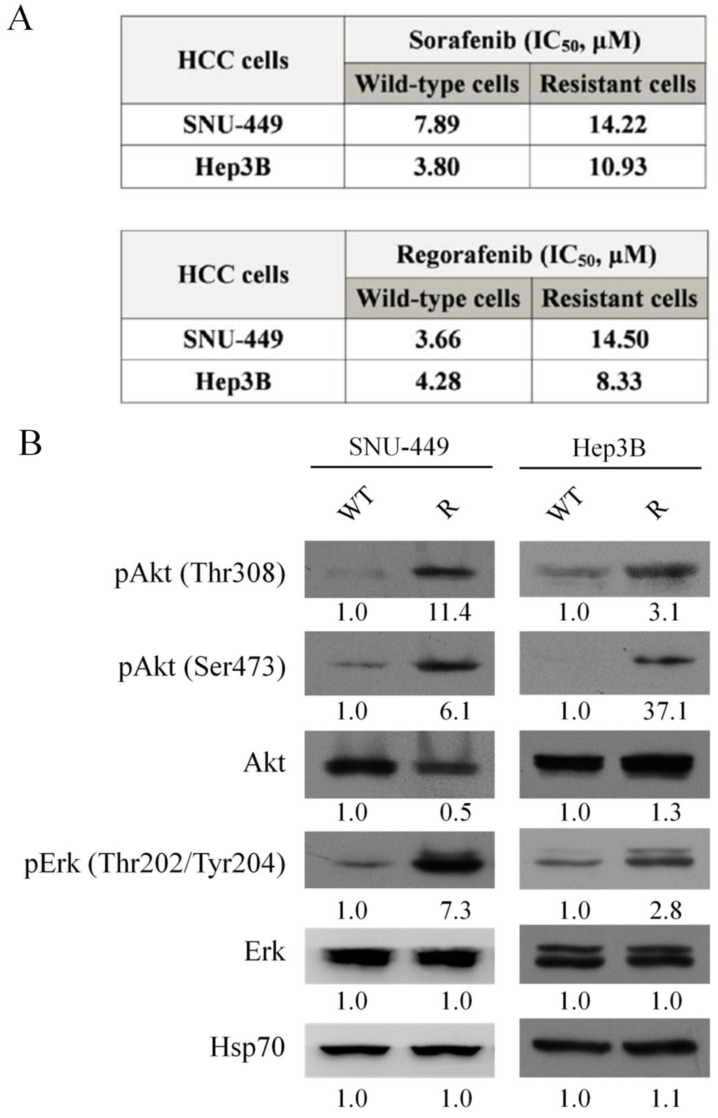
Establishment of sorafenib-resistant HCC cells. HCC cells were subjected to steadily increasing concentrations of sorafenib, with culture starting at 8.3 μM (IC_50_ for SNU-449 cells) or 3.7 μM (IC_50_ for Hep3B cells) sorafenib, and exposure dose increasing by 0.5 μM each week: (**A**) IC_50_ of various cell lines responding to sorafenib or regorafenib. Cell viability was determined by MTS assay. (**B**) Expression of phosphorylated Akt, Akt, phosphorylated Erk, and Erk analyzed by western blotting in WT and sorafenib-resistant (R) cells. Numeric values listed below the bands represent the indicated protein expression by densitometry analysis relative to levels in the WT cells.

Long-term exposure of HCC cells to sorafenib leads to development of drug resistance. Previous studies have revealed several mechanisms involved in sorafenib resistance in liver cancer, including activation of PI3K/Akt pathways, hypoxia-inducible pathways, and epithelial-mesenchymal transition [20]. We established sorafenib-resistant cell lines in order to examine mechanisms of sorafenib resistance in HCC cell lines. SNU-449 and Hep3B cells were exposed to steadily increasing concentrations of sorafenib. Sorafenib-resistant cells were selected and found to be more resistant to sorafenib-induced cell death (IC_50_ = 14.22/10.93 μM) than control cells (IC_50_ = 7.89/3.80 μM) (Figure 2A, upper panel). Regorafenib, a structural analogue of sorafenib, was approved in 2018 as a second-line treatment for patients in whom HCC progresses despite sorafenib treatment [21]. We treated sorafenib-resistant cell lines with regorafenib and determined cell viability by colorimetric MTT assay. Sorafenib-resistant cells were also more resistant to regorafenib-induced cell death (IC_50_ = 14.50/8.33 μM) than control cells (IC_50_ = 3.66/4.28 μM) (Figure 2A, lower panel). Phosphorylated Akt and phosphorylated Erk showed higher expression in sorafenib-resistant cells (Figure 2B). These findings suggest that overactivation of PI3K/Akt signal transduction cascade is involved in sorafenib resistance in HCC cells.

### 3.3. Regulation of Multidrug-Resistant Proteins by USP22 in Sorafenib-Resistant Cells

**Figure 3:**
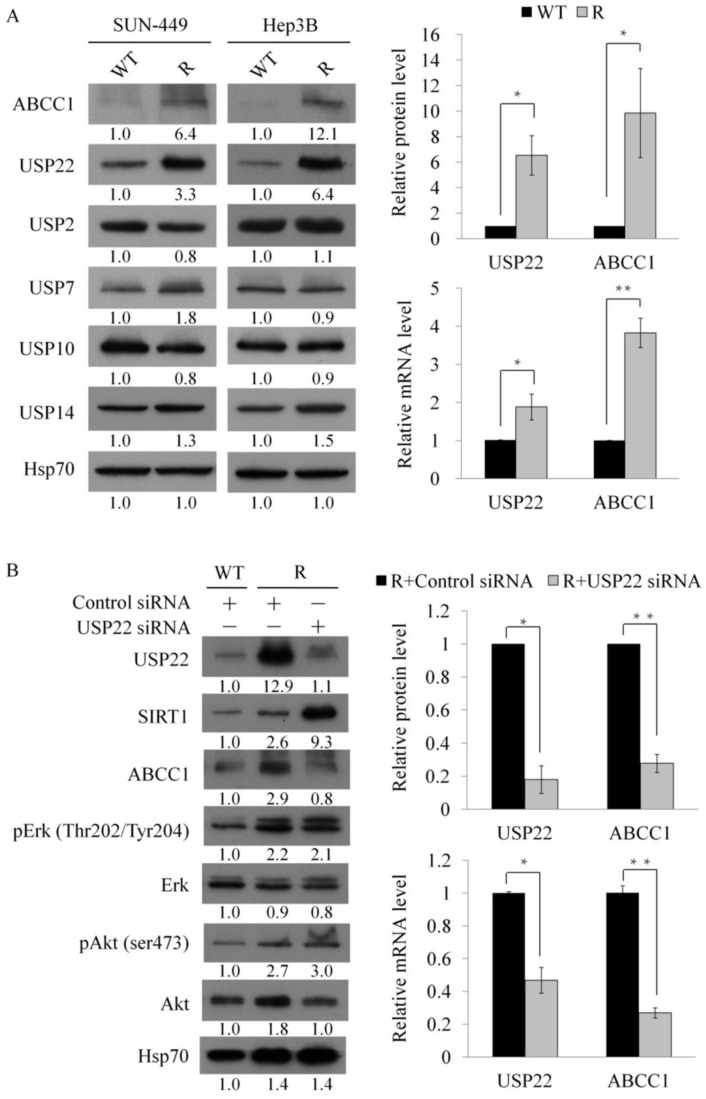
Regulation of multidrug-resistant proteins by USP22 in sorafenib-resistant HCC cells: (**A**) Expression of USPs analyzed by western blotting and RT-qPCR in WT and sorafenib-resistant (R) cells. *: *p* < 0.05; **: *p* < 0.01 compared with WT group. (**B**) Expression of multidrug-resistant proteins in USP22 knockdown and sorafenib-resistant Hep3B cells analyzed by western blotting and RT-qPCR. Numeric values listed below the bands represent the indicated protein expression by densitometry analysis relative to levels in the WT cells. *: *p* < 0.05; **: *p* < 0.01 compared with control group.

Overexpression of multidrug-resistant proteins has been reported to promote development of sorafenib resistance [22]. However, little is known regarding regulation of multidrug-resistant proteins in sorafenib-resistant HCC. Aberrant expression of USPs is strongly correlated with multidrug resistance and cancer progression. We examined expression of USPs by western blotting to clarify the role of USPs in development of sorafenib resistance in HCC. In sorafenib-resistant cells, the increase in expression for USP22 was significantly greater than that for other USPs (USP2, USP7, USP10, and USP14) (Figure 3A). Expression of multidrug resistance-associated protein 1 (MRP1/ABCC1) was also upregulated in these cells.

USP22 has been reported to promote ABCC1 expression in HCC cells by activating the SIRT1/Akt/MRP1 pathway [23]. To investigate the relationship between USP22 and multidrug resistance proteins, we infected sorafenib-resistant HCC cells with lentiviruses expressing siRNA that targets USP22. Treatment of these cells with USP22 siRNA significantly reduced endogenous USP22 and ABCC1 protein and mRNA expression levels (Figure S1). USP22 directly regulates ABCC1 expression at the transcriptional level (Figure 3B and Figure S2). Thus, our findings indicate that USP22 regulates ABCC1 expression in HCC cells and promotes sorafenib resistance. Treatment of these cells with USP22 siRNA did not notably alter the expression of phosphorylated Akt or phosphorylated Erk. However, the expression of SIRT1 was dramatically increased after knockdown of USP22 in sorafenib-resistant HCC cells. It could be a compensatory response for decreasing USP22 and an attempt by the HCC cells to maintain the sorafenib resistance.

USP family members contain highly conserved catalytic domains and usually form macromolecular complexes with ubiquitin ligase. To investigate the relationship between other USPs and ABCC1, we also infected sorafenib-resistant HCC cells with lentiviruses expressing siRNA that targets USP10 and USP14 (Figure S3). Knockdown of USP10 or USP14 did not decrease the expression of ABCC1. Moreover, it appears that the expressions of USP10 might compensate for the knockdown effect of USP14 and vice versa.

### 3.4. Correlation of USP22 and ABCC1 Expression in Sorafenib-Resistant HCC Tissues

**Figure 4:**
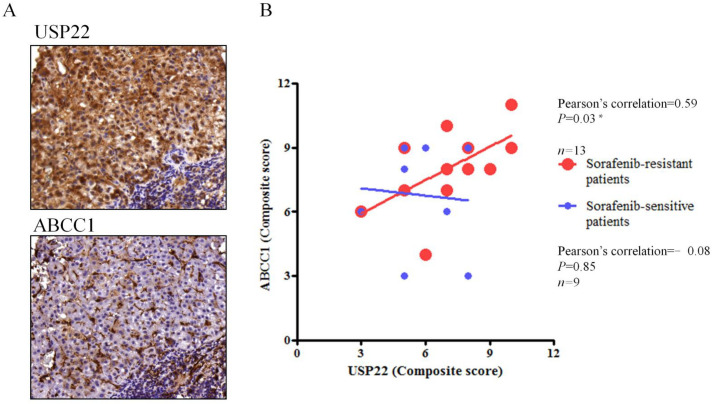
Positive correlation of USP22 and ABCC1 expression levels in sorafenib-resistant HCC tissues. (**A**) USP22 and ABCC1 expression detected by IHC staining of primary HCC. (**B**) Positive correlation between USP22 (*x*−axis) and ABCC1 (*y*−axis) H-scores based on IHC. *: *p* < 0.05 compared with sorafenib-sensitive patients group.

Regulation of ABCC1 expression by USP22 in sorafenib-resistant HCC cells was indicated by the above findings; however, the role of these molecules in sorafenib resistance in a clinical context remained unclear. We used IHC staining to examine expression of USP22 and ABCC1 in 13 sorafenib-resistant and 9 sorafenib-sensitive HCC tissue samples. Expression of the two molecules positively correlated with each other in sorafenib-resistant samples, but not in sorafenib-sensitive samples (Figure 4). Therefore, upregulation of USP22 and ABCC1 expression during sorafenib treatment can potentially serve as poor response markers, and USP22 is a potential therapeutic target for overcoming sorafenib resistance.

## 4. Discussion

Various mechanisms involved in development of sorafenib resistance have been suggested by recent studies, including alterations of PI3K/Akt and RAF/MEK/Erk pathways [24]. Sorafenib resistance in HCC may also be modulated through expression of EGFR, HIF-1α, HIF-2α, long noncoding RNAs, and microRNAs [25,26,27,28]. Sorafenib resistance can be reduced or overcome using inhibitors of CDK1, MEK, or receptor tyrosine kinases [29,30,31,32,33]. Selective small-molecule inhibitors have been approved by the US FDA for clinical treatment of certain cancers; however, they present a variety of adverse side effects. Deregulated MicroRNAs may also mediate drug resistance, and some of them are currently proceeding to clinical trial phase; however, technical challenges of this approach in clinical settings include problems with stability, delivery, and triggering of immune responses and some potential untoward effects because microRNA may have multiple target genes. Development of new therapeutic approaches for overcoming sorafenib resistance has been a high priority in HCC research.

High USP22 expression was observed in HCC patients, and USP22 knockdown enhanced chemosensitivity to 5-FU in HCC cells [34]. SIRT1 physically interacts with USP22 as a mediator of USP22. Our results revealed the positive correlation among USP22, SIRT1, and ABCC1 expression that were observed in all 6 HCC cell lines. Sorafenib-resistant cells with USP22 siRNA significantly induced endogenous SIRT1 expression and could be a compensatory response to maintain the sorafenib resistance. However, the increasing of SIRT1 did not rescue ABCC1 expression. It indicated that USP22 plays a dominant role in sorafenib resistance.

In normal culture conditions, basal levels of ABCC1, USP22, and SIRT1 did not show a correlation to sorafenib sensitivity both in well differentiated and poorly differentiated cell lines. Interestingly, the expression of ABCC1, USP22, and SIRT1 were upregulated in sorafenib-resistant cells compared to those in basal status of wild-type cells. Taking together these findings, USP22 might be maintained at relatively low levels in malignant HCC cells and is markedly increased under long-term sorafenib treatment as a strategy to overcome cell death induced by sorafenib treatment. The detail mechanism of sorafenib mediated USP22 induction still needs further investigation. These findings are supported by the findings that USP22 correlates with ABCC1 expression in sorafenib-resistant HCC samples, but not in sensitive HCC samples.

Aberrant expression of various USPs can serve as a tumor biomarker and is often strongly correlated with cancer progression. USP10 promotes proliferation in HCC [35]. USP14 expression in HCC is significantly elevated in tumor tissues and promotes tumor progression [36]. Findings of the present study demonstrate that USP22 is overexpressed in sorafenib-resistant SNU-449 and Hep3B cells, and that ABCC1 expression in these cells is strongly reduced by USP22 knockdown. However, knockdown of USP10 or USP14 did not decrease the expression of ABCC1. USP22 plays a key role in regulating expression of multidrug-resistant proteins. Taken together, the upregulation of ABCC1 expression by increased USP22 in sorafenib resistance is specific.

High USP22 expression levels occur in various cancers and have the potential application as predictive or prognostic markers [37]. Under hypoxic conditions, USP22 promotes HCC stemness through an HIF1α feedback loop [38]. High USP22 and SIRT1 expression in HCC patients was correlated with 5-FU resistance [39]. Reduction of USP22 expression decreased tumorigenicity and enhanced anticancer drug sensitivity [40]. In the present study, sorafenib-resistant HCC samples showed elevated USP22 expression. A positive correlation between USP22 and ABCC1 expression was observed in sorafenib-resistant HCC tissues. USP22 regulated ABCC1 expression and was associated with sorafenib resistance. Targeting of USP22 is a promising new strategy to overcome drug resistance in cancer therapy. USP22 knockdown did not notably affect pErk or pAkt expression (Figure 3B), suggesting the existence of different mechanisms of sorafenib resistance.

Numerous USP inhibitors have been identified and can potentially be developed as anticancer agents. Wu-5 or Spautin-1 had inhibitory effects on USP10, and USP14 was inhibited by WP1130 and B-AP15 [41,42]. Only a few USP22 inhibitors have been reported. Future studies will focus on development of new USP22 inhibitors and characterization of upstream USP22 signaling mechanisms.

## 5. Conclusions

Our present findings demonstrate significantly elevated USP22 and ABCC1 expression in sorafenib-resistant cells. Knockdown of endogenous USP22 by specific siRNA strongly reduced ABCC1 expression in these cells. USP22 regulated ABCC1 expression, which was correlated with sorafenib resistance. Correlated expression of USP22 and ABCC1 was also observed in tissues of sorafenib-resistant HCC patients, consistently with in vitro findings. Thus, USP22 has strong potential as a therapeutic target to overcome sorafenib resistance in HCC patients.

## Data Availability

The data underlying this article will be shared on reasonable request to the corresponding author.

## Supplementary Materials

The following supporting information can be downloaded at: https://www.mdpi.com/article/10.3390/cells11040634/s1, Figure S1: Protein levels in USP22 knockdown cells. Figure S2: Expression of multidrug-resistant proteins in USP22 knockdown cells analyzed by western blotting. Figure S3: Knockdown of USP10 or USP14 did not decrease the expression of ABCC1.
