# Supplementary material for: Upregulation of USP22 and ABCC1 during Sorafenib Treatment of Hepatocellular Carcinoma Contribute to Development of Resistance
Source: Cells. 2022 Feb 11;11(4):634. doi: 10.3390/cells11040634 (PMC8870465; doi:10.3390/cells11040634)
Supplement: Supplementary file 1 [file cells-11-00634-s001.zip › cells-1551992-supplementary.pdf]

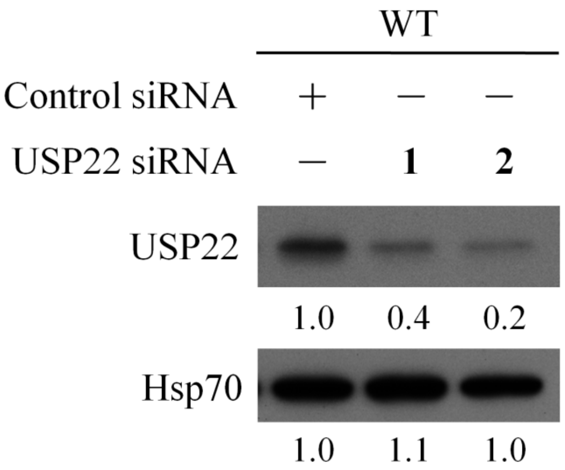

Figure S1. Protein levels in USP22 knockdown cells. Expression of USP22 proteins in Hep3B cells analyzed by western blotting. Numeric values listed below the bands represent of indicated protein expression by densitometry analysis relative to levels in the control siRNA cells.

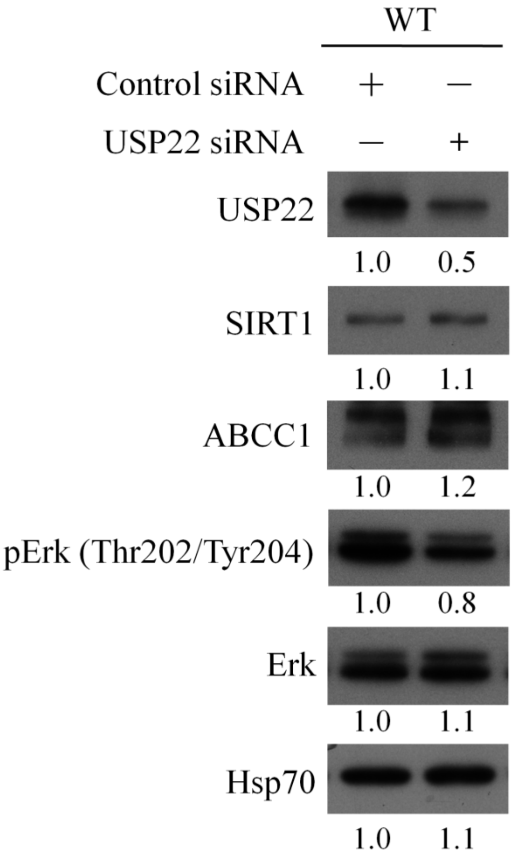

Figure S2. Expression of multidrug-resistant proteins in USP22 knockdown cells analyzed by western blotting. Numeric values listed below the bands represent of indicated protein expression by densitometry analysis relative to levels in the control siRNA cells.

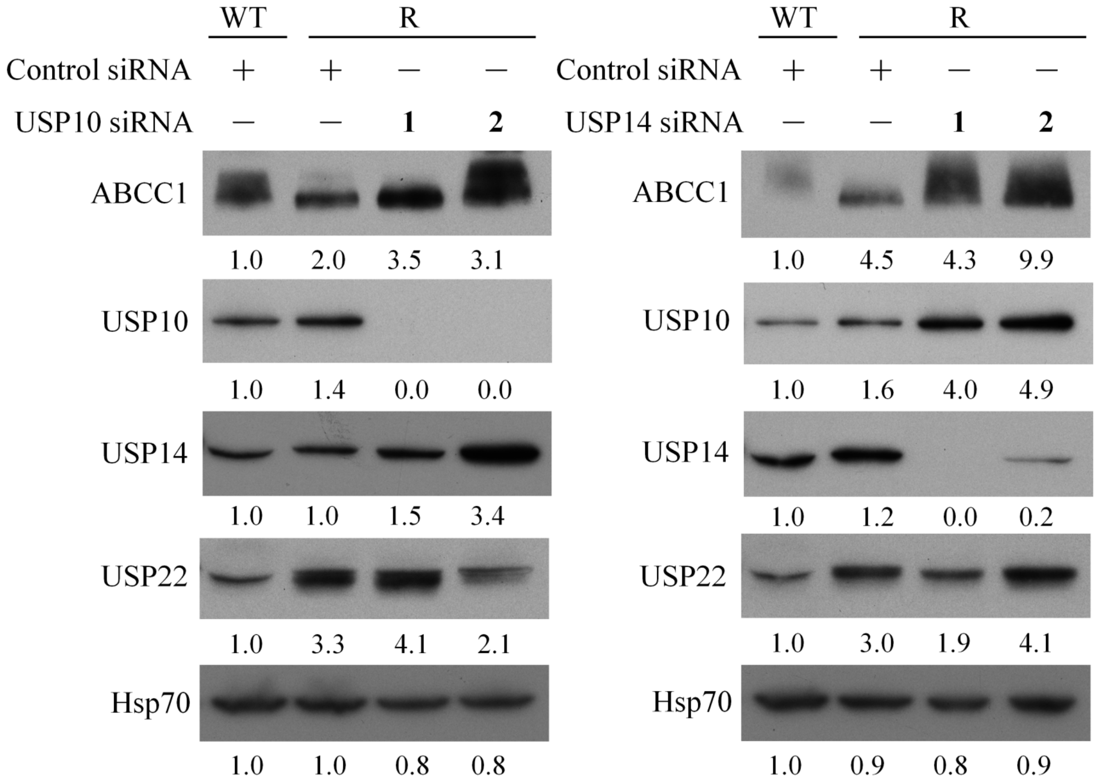

Figure S3. Knockdown of USP10 or USP14 did not decrease the expression of ABCC1. Expression of ABCC1 and USPs in USP10 or USP14 knockdown and sorafenib-resistant cells analyzed by western blotting. Numeric values listed below the bands represent of indicated protein expression by densitometry analysis relative to levels in the WT cells.
